# Judicialization and right to health in Brazil: a trajectory of matches and mismatches

**DOI:** 10.11606/s1518-8787.2023057004579

**Published:** 2023-02-08

**Authors:** Fabiola Sulpino Vieira

**Affiliations:** I Instituto de Pesquisa Econômica Aplicada Diretoria de Estudos e Políticas Sociais Brasília DF Brasil Instituto de Pesquisa Econômica Aplicada. Diretoria de Estudos e Políticas Sociais. Brasília, DF, Brasil

**Keywords:** Health’s Judicialization, Access to Essential Medicines and Health Technologies, Equity in the Resource Allocation, Unified Health System, Right to Health

## Abstract

This study discusses the impacts of judicialization on the guarantee of the right to health in Brazil and the need to reassess the role of the Judicial system in its protection. We used evidence from the technical-scientific literature and information on the budgetary-financial execution and the acquisition of medicines from the Brazilian Ministry of Health to substantiate the arguments. In 2019, lawsuits consumed 25.2% of the resources of the Specialized Component of Pharmaceutical Care, 21% for 10 medicines. Although the Judicial promotes this right when the State fails to ensure access to medicines incorporated into the Brazilian Unified Health System (SUS), this system compromises access to medicines of the population with the determinations of acquisition of non-incorporated products. The Judicial needs to guide its control over compliance with constitutional and legal precepts in public policies, especially in fiscal policy, given its impact on the financing of the SUS.

## INTRODUCTION

In Brazil, lawsuits in the health field became more important in recent decades due to the significant increase in cases and their impacts, especially for the Brazilian Unified Health System (SUS). The judicialization of health can be understood as a situation of expansion of the activation of the Judicial by individuals or groups of individuals, citizens or consumers, to arbitrate conflicts of this system with the Executive Branch, with private companies and individuals in healthmatters^1^.

In cases where the defendant is the State, the impact of judicialization on the guarantee of the right to health is positive or negative. On one hand, judicialization would be beneficial because it constitutes a means to ensure the right to health and to induce improvement in the response of the State^2^. On the other hand, judicialization would produce unequal treatment among citizens in a country marked by great socioeconomic inequalities and health inequities^3,4^.

In the SUS, lawsuits demand medicines^5^ due to failures in the supply of incorporated products and request experimental or approved medicines for commercialization, but not incorporated into the system^4,7^. These situations may be prevalent, depending on the locality, and are important in assessing the impacts of health judicialization.

Thus, considering the relevance of this theme, this study aimed to discuss the impacts of the current model of judicialization on the guarantee of the right to health in Brazil and the need to reevaluate the role of the Judicial in the protection of this right.

We obtained evidence from the technical-scientific literature on the judicialization of health and the financing of the SUS by research done in the Virtual Health Library, in the field “title, abstract and subject”, for documents published since 2000, including all bases and using the words: i) “judicialization” and “health”; (ii) “financing” and “SUS”. We also consulted references on these topics in documents and publications of the National Council of Justice (CNJ). We selected studies addressing the following themes: consequences of lawsuits for health policy, theory of the reservation of the possible, and role of the Judicial in the protection of social rights.

Data from the following information systems were also obtained to substantiate the arguments: i) Justice in Numbers Panel of CNJ: new cases of judicial demand (2014 to 2020); ii) Integrated General Services Administration System (SIASG): acquisition of medicines by the Brazilian Ministry of Health (MS) (2016-2020); and iii) *Siga Brasil* tool: budget-financial execution of the MS (2012-2020).

### Positive and Negative Consequences of Judicialization

The judicialization of public health began in the 1990s with lawsuits that demanded treatment for HIV-positive people. Decisions in favor of patients represented an advance in ensuring universal and integral access to health services and goods^8^. Since then, the demands have diversified and multiplied, mostly individually, favoring the perception that, although part of them is relevant to ensure the right to health, another part has the potential to disorganize the SUS^9^.

**Figure 1:**
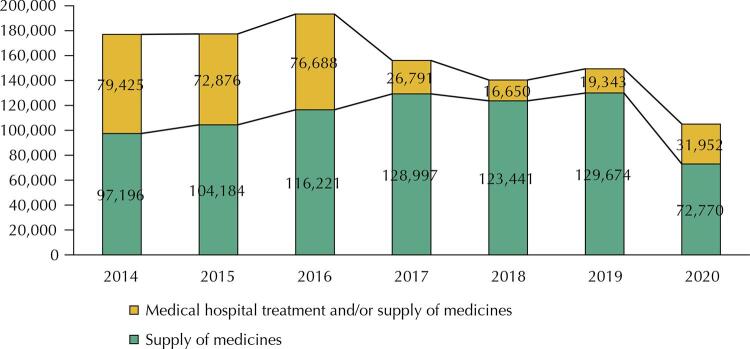
New cases of lawsuits involving medicines (2014–2020). Note: query according to subject classification number 4, which is used for indexing processes.

Source: National Council of Justice. Justice in Numbers Panel. Available from: <https://bit.ly/3GfXk9O>. Accessed on: Jan 25, 2022.

Several institutions implemented measures to broaden the dialogue between the systems and establish beacons for judicial decisions. However, despite the efforts made^1^, the new cases did not decrease (Figure 1). The year of 2020 is atypical because of the negative impacts of the COVID-19 pandemic on the demand and supply of health services^10^ and other public services, including those of the justice system.

The statements of the CNJ, published since 2014 as guidelines to magistrates in the face of the judicialization of health^11^, also appeared ineffective. From 2008 to 2017, the mention of statements was of 0.02% in first instance decisions and less than 0.01% in second instance decisions^5^.

Among the judicialized items acquired by the Brazilian Ministry of Health from 2016 to 2020, most of the 10 medicines with the highest budgetary impact were not incorporated into the SUS (Table). Some decisions also determined the purchase of medicines without registration with the National Health Surveillance Agency (Anvisa), opposite to what guides the statement 50^11^.

**Table:** Expenditure of the Ministry of Health on the acquisition of medicines by judicial determination (2015–2020).

Year	Judicialized medicines		10 medicines with the greatest budgetary impact					
	Number of items	Total expenditure (R$ of 2020)	Name	Incorporation	Indication	Expenditure on the item (R$ of 2020)	Expenditure on ten medicines (R$ 2020)	% of spending on the ten medicines in the total spending
2016	676	1,436,444,910	1. eculizumab 10 mg/ml	2018 (Ordinance no. 77, of 12/14/2018)	Paroxysmal nocturnal hemoglobinuria	717,149,490	1,307,172,609	91.0
			2. galsulfase 1 mg/ml	2018 (Ordinance no. 83, of 12/19/2018)	Mucopolysaccharidosis type VI	145,918,651		
			3. elosulfase alfa 1 mg/ml	2018 (Ordinance no. 82, of 12/19/2018)	Mucopolysaccharidosis type IVA (Morquio Syndrome A)	107,462,354		
			4. galsulfase 2 mg/ml	2017 (Ordinance no. 62, of 12/19/2017)	Mucopolysaccharidosis type II	83,442,677		
			5. alfagalsidase 1 mg/ml	Not incorporated (decision not to incorporate Ordinance no. 56, of 11/23/2020)	Fabry disease	80,920,978		
			6. ataluren 250 mg	Not incorporated (no evaluation by Conitec)	Duchenne Muscular Dystrophy	55,633,789		
			7. agalsidase beta 35 mg	Not incorporated (decision not to incorporate Ordinance No. 56, of 11/23/2020)	Fabry disease	37,717,391		
			8. metreleptin 11.3 mg	Not incorporated and without registration with Anvisa	Congenital or acquired generalized lipodystrophy	32,054,438		
			9. lomitapide 10 mg	Not incorporated (no evaluation by Conitec)	Familial hypercholesterolemia	23,927,105		
			10. human C1 esterase inhibitor 500 IU	Not incorporated (no evaluation by Conitec)	Hereditary angioedema type I and II	22,945,735		
2017	508	1,085,490,298	1. eculizumab 10 mg/ml	2018 (Ordinance No. 77, of 12/14/2018)	Paroxysmal nocturnal hemoglobinuria	296,462,726	981,720,251	90.4
			2. galsulfase 1 mg/ml	2018 (Ordinance No. 83, of 12/19/2018)	Mucopolysaccharidosis type VI	176,252,001		
			3. elosulfase alfa 1 mg/ml	2018 (Ordinance No. 82, of 12/19/2018)	Mucopolysaccharidosis type IVA (Morquio Syndrome A)	101,093,948		
			4. alfagalsidase 1 mg/ml	Not incorporated (decision not to incorporate Ordinance No. 56, of 11/23/2020)	Fabry disease	100,592,156		
			5. ataluren 250 mg	Not incorporated (no evaluation by Conitec)	Duchenne Muscular Dystrophy	84,746,590		
			6. idursulfase 2 mg/ml	2017 (Ordinance No. 62, of 12/19/2017)	Mucopolysaccharidosis type II	61,684,075		
			7. agalsidase beta 35 mg	Not incorporated (decision not to incorporate Ordinance No. 56, of 11/23/2020)	Fabry disease	54,882,175		
			8. ataluren 1000 mg	Not incorporated (no evaluation by Conitec)	Duchenne Muscular Dystrophy	39,848,443		
			9. alpha-glucosidase 50 mg	2019 (Ordinance No. 48, 10/16/2019)	Pompe disease	33,258,882		
			10. metreleptin 11.3 mg	Not incorporated and without registration with Anvisa	Congenital or acquired generalized lipodystrophy	32,899,255		
2018	332	1,495,905,549	1. eculizumab 10 mg/ml	2018 (Ordinance No. 77, of 12/14/2018)	Paroxysmal nocturnal hemoglobinuria	478,746,855	1,364,024,905	91.2
			2. galsulfase 1 mg/ml	2018 (Ordinance No. 83, of 12/19/2018)	Mucopolysaccharidosis type VI	164,038,893		
			3. idursulfase 2 mg/ml	2017 (Ordinance No. 62, of 12/19/2017)	Mucopolysaccharidosis type II	124,079,026		
			4. nusinersen 2.4 mg/ml	2021 (Ordinance No. 26, of 6/1/2021)	Spinal muscular atrophy 5q type II, diagnosed up to 18 months of age	124,045,961		
2018	332	1,495,905,549	5. elosulfase alfa 1 mg/ml	2018 (Ordinance No. 82, of 12/19/2018)	Mucopolysaccharidosis type IVA (Morquio Syndrome A)	112,287,809	1,364,024,905	91.2
			6. alfagalsidase 1 mg/ml	Not incorporated (decision not to incorporate Ordinance No. 56, of 11/23/2020)	Fabry disease	105,496,952		
			7. ataluren 250 mg	Not incorporated (no evaluation by Conitec)	Duchenne Muscular Dystrophy	100,947,361		
			8. ataluren 1,000 mg	Not incorporated (no evaluation by Conitec)	Duchenne Muscular Dystrophy	55,002,927		
			9. agalsidase beta 35 mg	Not incorporated (decision not to incorporate Ordinance No. 56, of 11/23/2020)	Fabry disease	53,825,639		
			10. alpha-glucosidase 50 mg	2019 (Ordinance No. 48, 10/16/2019)	Pompe disease	45,553,481		
2019	284	1,004,114,249	1. eculizumab 10 mg/ml	2018 (Ordinance No. 77, of 12/14/2018)	Paroxysmal nocturnal hemoglobinuria	467,571,795	950,325,051	94.6
			2. elosulfase alfa 1 mg/ml	2018 (Ordinance No. 82, of 12/19/2018)	Mucopolysaccharidosis type IVa (Morquio Syndrome A)	190,076,099		
			3. alfagalsidase 1 mg/ml	Not incorporated (decision not to incorporate Ordinance No. 56, of 11/23/2020)	Fabry disease	105,003,061		
			4. nusinersen 2.4 mg/ml	2021 (Ordinance No. 26, of 6/1/2021)	Spinal muscular atrophy 5q type II	87,703,893		
			5. galsulfase 1 mg/ml	2018 (Ordinance No. 83, of 12/19/2018)	Mucopolysaccharidosis type VI	54,288,525		
			6. metreleptin 11.3 mg	Not incorporated and without registration with Anvisa	Congenital or acquired generalized lipodystrophy	21,533,960		
			7. eteplirsen 50 mg/ml	Not incorporated and without registration with Anvisa	Duchenne Muscular Dystrophy	8,324,480		
			8. sebelipase alfa 2 mg/ml	Not incorporated (no evaluation by Conitec)	Lysosomal acid lipase deficiency	6,308,632		
			9. brentuximab vedotin 50 mg	2019 (Ordinance No. 12, of 3/11/2019)	Hodgkin’s lymphoma cd30+	5,976,991		
			10. mercaptamine 75 mg	Not incorporated and without registration with Anvisa	Nephropathic cystinosis	3,537,616		
2020	678	592,320,679	1. eculizumab 10 mg/ml	2018 (Ordinance No. 77, of 12/14/2018)	Paroxysmal nocturnal hemoglobinuria	220,640,260	484,460,069	81,8
			2. ataluren 250 mg	Not incorporated (no evaluation by Conitec)	Duchenne Muscular Dystrophy	107,559,900		
			3. clozapine 100 mg	2015 (Ordinance No. 3, of 3/9/2015) and 2016 (Ordinance No. 22, of 5/31/2016)	Bipolar affective disorder and psychosis associated with Parkinson’s disease	42,825,048		
			4. metreleptin 11.3 mg	Not incorporated and without registration with Anvisa	Congenital or acquired generalized lipodystrophy	36,465,099		
			5. alpha-glucosidase 50 mg	2019 (Ordinance No. 48, 10/16/2019)	Pompe disease	19,945,651		
			6. ataluren 1,000 mg	Not incorporated (no evaluation by Conitec)	Duchenne Muscular Dystrophy	19,580,225		
			7. ataluren 125 mg	Not incorporated (no evaluation by Conitec)	Duchenne Muscular Dystrophy	15,212,043		
			8. mercaptamine 75 mg	Not incorporated and without registration with Anvisa	Nephropathic cystinosis	10,014,300		
			9. sebelipase alfa 2 mg/ml	Not incorporated (no evaluation by Conitec)	Lysosomal acid lipase deficiency	8,655,498		
			10. cerliponase alfa 30 mg/ml	Under analysis by Conitec	Neuronal ceroid lipofuscinosis type 2	3,562,045		

Conitec: National Commission for the Incorporation of Technologies in the Brazilian Unified Health System.

Sources: i) Integrated General Services Administration System (SIASG). Report available by a technician from the Brazilian Ministry of Health; (ii) Conitec.

Features: Demanded technologies and Recommendations of Conitec. Available from: <http://conitec.gov.br/>. Accessed on: Jan 25, 2022.

Note: the same medicine, but at different concentrations, was counted as two or more drugs. Example: ataluren.

**Figure 2:**
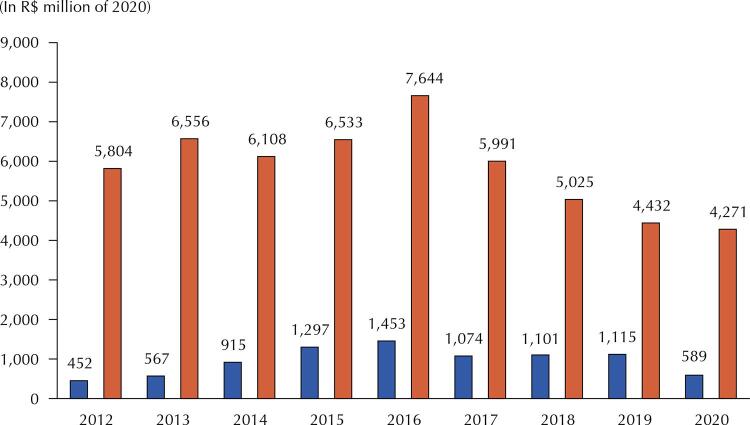
Expenditure of the Ministry of Health on medicine lawsuits and with the Specialized Component of Pharmaceutical Care - CEAF (2012–2020). Note: Expenditure in lawsuits refers to the settled expenditure, recorded in the Responsible Management Unit 250925-SJ-MED/MS. Expenditure on CEAF relates to the settled expenditure, recorded in budget action 4705–Financial Support for The Acquisition and Distribution of Medicines of the Specialized Component of Pharmaceutical Care.

Source: *Siga Brasil*. Available from: <https://bit.ly/3g50ClC>. Accessed on: Jan 25, 2022.

The Brazilian Ministry of Health’s expenditure on medicines lawsuits increased significantly between 2012 and 2016 (221%), reaching R$ 1.5 billion last year. It decreased 26% between 2016 and 2017 and remained at R$ 1. billion from 2017 to 2019 (Figure 2).

Studies in the 2000s already showed concern about the consequences of lawsuits. They explained^12,13^ issues such as the disregard of the responsibilities of the federation entities in the organization of the SUS, the purchase of medicines not incorporated and without registration with Anvisa, and losses to equity.

In the last decade, authors linked to the justice system entered the debate, highlighting positive and negative aspects of the judicialization of health, which represents an advance by favoring the discussion on the subject among their peers.

Regarding the positive aspects, we highlight the promotion of the formulation and review of public policies, the inclusion of health in the political agenda, the development of the evaluation of health technologies, and the expansion of dialogue between the powers^14,15^.

Regarding the negative aspects, we highlight the disorganization of the SUS and public finances, the undue judicial choices of public policies, the weakening of isonomy, the disregard of the criteria for prioritizing the technologies available, and the expansion of health inequalities^3,4,15^.

Moreover, the Judicial can consider scientific evidence and determine the supply of technologies not incorporated into the SUS, in a parallel and capillary process throughout the country, which competes with the evaluation of technologies performed by the health system^1^.

This understanding has several implications. Two implications stand out: i) the weakening of policies as a means for guaranteeing the right to health because, with so many exceptions to the National List of Essential Medicines (Rename), the rule, which should be valid for all, is distorted; and (ii) the reduction of the resources provided in the budget of the year to ensure the population’s access to Rename medicines. This second implication is discussed below.

### Reservation of the Possible, Judicialization, and Public Budget

Regarding the relationship between the public budget, the judicialization of health, and the theory of the reservation of the possible, the manifestations of public managers and magistrates are opposed. On one hand, managers use this theory to justify the impossibility of meeting the judicial demand, claiming the unavailability of resources to meet it. On the other hand, judges counter-argue that a secondary interest of the State cannot be superimposed on the right to health under the argument of the reservation of the possible. Both positions need to be rethought.

The concept of reservation of the possible came from Germany, where they recognized that issues involving social rights suffer limitation in three dimensions: i) reservation of the factually possible: the satisfaction of demand needs to be feasible; (ii) reservation of the legally possible: the demand needs to be legally possible; and iii) reservation of the financially possible: the demand fulfillment is limited to the state’s financial capacity^16^.

Regarding the use of the theory of the reservation of the possible by managers, financial capacity of the State is different from the annual budget established for health. The financial capacity of the State is measured considering all the resources collected from society. Revenues limit the capacity to spend on policies that empower social rights, thus society’s participation in the discussion conducted by its representatives on the allocation of resources is fundamental^17^. But a shortage of resources considering only the annual health budget cannot be claimed.

Regarding the counter-argument of the magistrates, the financial capacity cannot be considered as a secondary interest of the State. The rights have costs, thus is necessary to measure them and define how they will be financed, as well as control who decides on the resources that will be allocated to realize them^18^.

When the Judicial ignores the macro-issues related to the subject and determines the supply, for an individual, of medicines not provided for in public policies, it impacts the access of others to the medicines included in the policies^19^. This occurs because the budget has planning nature and is defined in the year prior to its validity. The reallocation of resources between different areas requires prior legislative authorization, not only the simple will of the health manager^20^ and, depending on the economic conditions of the country, the budget constraint may imply tragic choices in the supply of goods and services.

**Figura 3:**
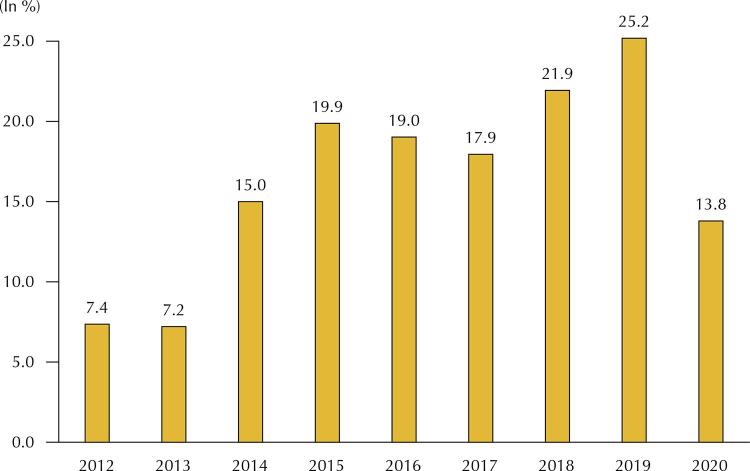
Participation of the Brazilian Ministry of Health’s expenditure in medicines lawsuits in the total expenditure on the Specialized Component of Pharmaceutical Care (2012–2020). Note: settled expense recorded in the Responsible Management Unit 250925-SJ-MED/MS.

Source: *Siga Brasil*. Available from: <https://bit.ly/3g50ClC>. Accessed on: Jan 25, 2022.

Figures 2 and 3 illustrate this type of problem well. In the Brazilian Ministry of Health, since 2014, resources allocated in the budget action that finance the Specialized Component of Pharmaceutical Assistance (CEAF) also finance the expenses with medicines lawsuits. Considering only the expenditure recorded in this action, the share of expenses for lawsuits has grown since 2012, reaching 25.2% in 2019. As a result, fewer resources finance the list of medicines of this component for the entire population.

Only ten drugs accounted for 94.6% of the expenditure on lawsuits from the Brazilian Ministry of Health in 2019 (R$ 950.33 million) and consumed 21% of the resources allocated to CEAF. Thus, there is a lack of medicines under the responsibility of acquisition of the Ministry of Health^21,22^, which should have several determinants, but certainly one of them is judicialization.

This example explains how judicialization can cause more harm than promoting the right to health in Brazil. Decisions on resource allocation are complex and require democratic legitimacy. Moreover, they require solid technical knowledge in health. Decisions cannot have as a single foundation criteria denoting humanitarian feelings of solidarity, justice, and empathy for a single individual^23^. Because this leads to decisions that disregard the country’s health legislation and that put people’s health at risk, as in the case of the phosphoethanolamine^24^. Finally, how could the Judicial better protect the right to health?

### Need for New Directions

The Judicial plays an important role by determining that the State fulfills its duty to ensure the supply of medicines incorporated into the SUS, in compliance with the guidelines and regulations of public policies. In a recent survey, 46% of magistrates said that they did not observe guidelines and regulations^6^.

Meanwhile, fiscal policy decisions are taken within the Union with a great negative impact on social rights, such as the implementation of the spending ceiling for primary expenditures, the freezing of minimum applications in health and education, the absence of limitation of financial expenses, and the expansion of tax expenditure^25^.

Given the importance of fiscal policy to guarantee rights, human rights principles for fiscal policy are discussed, aiming to approximate Economy and Law^26^. The Judicial needs to exercise macrojustice, which demands control over processes involving macroeconomic policies that affect the funding of SUS^1,27^. If the Judicial does not exercise macrojustice, it will continue to promote health inequities.

Thus, it is necessary to reflect on the role of the Judicial in the protection of social rights. According to Ferraz^28^ (2011), social rights would be adequately protected if the Judicial stopped interfering in the content of policies and began to act in the control of their formulation to ensure respect for constitutional and legal norms. Gebran Neto^15^ (2019) has the same position.

Therefore, the role of the Judicial in ensuring access to medicines incorporated into the SUS, respecting the standards established within the health system, is not questioned. If the norms and procedures are questionable, considering the goals of the Federal Constitution of 1988 and the laws, the norms and procedures are discussed. But it is unreasonable to allocate resources by lawsuits for the purchase of unincorporated medicines. These allocations mischaracterize the general rule that should be applied to all and drain scarce resources, while the SUS is subjected to a chronic underfunding process^29,30^. Therefore, universality is impaired and the right to health remains denied to the most socioeconomically disadvantaged.
