# Supplementary material for: Judicialization and right to health in Brazil: a trajectory of matches and mismatches
Source: Rev Saude Publica. 2023 Feb 8;57:1. doi: 10.11606/s1518-8787.2023057004579 (PMC9933639; doi:10.11606/s1518-8787.2023057004579)
Supplement: Translation

# Translation: Judicialização e direito à saúde no Brasil: uma trajetória de encontros e desencontros

**Authors:** Fabiola Sulpino Vieira

**Affiliations:** I Instituto de Pesquisa Econômica Aplicada Diretoria de Estudos e Políticas Sociais Brasília DF Brasil Instituto de Pesquisa Econômica Aplicada. Diretoria de Estudos e Políticas Sociais. Brasília, DF, Brasil

**Keywords:** Judicialização da Saúde, Acesso a Medicamentos Essenciais e Tecnologias em Saúde, Equidade na Alocação de Recursos, Sistema Único de Saúde, Direito à Saúde

## Abstract

Neste texto, discutem-se os impactos da judicialização na garantia do direito à saúde no Brasil e a necessidade de reavaliação do papel do Judiciário na sua proteção. Evidências da literatura técnico-científica e informações sobre a execução orçamentário-financeira e a aquisição de medicamentos do Ministério da Saúde foram utilizadas para fundamentar os argumentos. Mostra-se que, em 2019, as ações judiciais consumiram 25,2% dos recursos do Componente Especializado da Assistência Farmacêutica, sendo 21% para 10 medicamentos. Argumenta-se que, embora o Judiciário promova esse direito quando o Estado falha em assegurar o acesso a medicamentos incorporados ao Sistema Único de Saúde (SUS), ele compromete o acesso a medicamentos da população com as determinações de aquisição de produtos não incorporados. Defende-se a necessidade de o Judiciário pautar seu controle sobre a observância dos preceitos constitucionais e legais nas políticas públicas, especialmente na política fiscal, dado seu impacto sobre o financiamento do SUS.

## INTRODUÇÃO

No Brasil, as ações judiciais no campo da saúde ganharam importância nas últimas décadas em razão do aumento significativo dos casos e de seus impactos, especialmente para o Sistema Único de Saúde (SUS). A judicialização da saúde pode ser entendida como uma situação de ampliação do acionamento do Poder Judiciário por parte de indivíduos ou grupos de indivíduos, na condição de cidadãos ou de consumidores, a fim de que sejam arbitrados conflitos destes com o Poder Executivo, com empresas privadas e pessoas físicas em matéria de saúde^1^.

Nos casos em que o réu é o Estado, o impacto da judicialização na garantia do direito à saúde tem sido apontado como positivo ou negativo. Por um lado, a judicialização seria benéfica por constituir um meio para garantir o direito à saúde e induzir melhora na resposta do Estado^2^. Por outro lado, produziria tratamento desigual entre os cidadãos, em um país marcado por grandes desigualdades socioeconômicas e iniquidades em saúde^3,4^.

No SUS, as ações judiciais demandam medicamentos^5^^,^^6^ devido a falhas no fornecimento dos produtos incorporados e solicitam medicamentos experimentais ou aprovados para comercialização, mas não incorporados ao sistema^4^^,^^7^. Essas situações podem ser mais ou menos prevalentes, a depender da localidade, e são importantes na avaliação dos impactos da judicialização da saúde.

Assim, considerando a relevância desse tema, este texto tem por objetivo discutir os impactos do modelo atual de judicialização sobre a garantia do direito à saúde no Brasil e a necessidade de reavaliação do papel do Judiciário na proteção desse direito.

Evidências da literatura técnico-científica sobre a judicialização da saúde e o financiamento do SUS foram obtidas a partir de pesquisa feita na Biblioteca Virtual em Saúde, no campo “título, resumo e assunto”, para documentos publicados a partir do ano 2000, incluindo-se todas as bases e utilizando-se das palavras: i) “judicialização” e “saúde”; ii) “financiamento” e “SUS”. Também foram consultadas referências sobre esses temas em documentos levantados e publicações do Conselho Nacional de Justiça (CNJ). Foram selecionados trabalhos que abordassem os seguintes temas: consequências das ações judiciais para a política de saúde, teoria da reserva do possível e papel do Judiciário na proteção de direitos sociais.

Dados dos seguintes sistemas de informação também foram obtidos para fundamentar os argumentos: i) Painel Justiça em Números do CNJ: casos novos de demanda judicial (2014 a 2020); ii) Sistema Integrado de Administração de Serviços Gerais (SIASG): aquisição de medicamentos pelo Ministério da Saúde (MS) (2016-2020); e iii) Siga Brasil: execução orçamentário-financeira do MS (2012-2020).

### Consequências Positivas e Negativas da Judicialização

A judicialização da saúde pública teve início nos anos 1990 com as ações judiciais que demandavam tratamentos para pessoas HIV positivas. As decisões em favor dos pacientes representaram um avanço na garantia do acesso universal e integral aos serviços e bens de saúde^8^. Desde então, as demandas se diversificaram e multiplicaram, sendo majoritariamente individuais, favorecendo a percepção de que, embora parte delas seja relevante para assegurar o direito à saúde, outra parte tem potencial para desorganizar o SUS^9^.

Várias instituições implementaram medidas para ampliar o diálogo entre os poderes e estabelecer balizas para as decisões judiciais. Contudo, a despeito dos esforços empreendidos^1^, os casos novos não diminuíram (Figura 1). O ano de 2020 é atípico por causa dos impactos negativos da pandemia de covid-19 sobre a procura e a oferta de serviços de saúde^10^ e dos demais serviços públicos, entre eles, os do sistema de justiça.

**Figura 1:** Casos novos de ações judiciais envolvendo medicamentos (2014–2020).

Fonte: Conselho Nacional de Justiça. Painel Justiça em Números. Disponível em: <https://bit.ly/3GfXk9O>. Acesso em: 25 jan. 2022.

Nota: consulta segundo a classificação de assuntos número 4, que é utilizada para a indexação dos processos.

Também parecem ter sido pouco efetivos os enunciados do CNJ, publicados desde 2014 como orientações aos magistrados no enfrentamento da judicialização da saúde^11^. De 2008 a 2017, verificou-se que a menção aos enunciados foi de apenas 0,02% nas decisões de primeira instância e de menos de 0,01% nas de segunda instância^5^.

Entre os itens judicializados adquiridos pelo Ministério da Saúde de 2016 a 2020, a maioria dos 10 medicamentos de maior impacto orçamentário não estava incorporada ao SUS (Tabela). Houve também decisões que determinaram a compra de medicamentos sem registro na Agência Nacional de Vigilância Sanitária (Anvisa), em contrário ao que orienta o enunciado 50^11^.

**Tabela:** Gasto do Ministério da Saúde com a aquisição de medicamentos por determinação judicial (2015–2020).

Ano	Medicamentos judicializados		Dez medicamentos de maior impacto orçamentário					
	Número de itens	Gasto total (R$ de 2020)	Nome	Incorporação	Indicação	Gasto com o item (R$ de 2020)	Gasto com os 10 medicamentos (R$ de 2020)	% do gasto com os 10 medicamentos no gasto total
2016	676	1.436.444.910	1. eculizumabe 10 mg/ml	2018 (Portaria nº 77, de 14/12/2018)	Hemoglobinúria paroxística noturna	717.149.490	1.307.172.609	91,0
			2. galsulfase 1 mg/ml	2018 (Portaria nº 83, de 19/12/2018)	Mucopolissacaridose tipo VI	145.918.651		
			3. elosulfase alfa 1 mg/ml	2018 (Portaria nº 82, de 19/12/2018)	Mucopolissacaridose tipo IVa (Síndrome de Morquio A)	107.462.354		
			4. idursulfase 2 mg/ml	2017 (Portaria nº 62, de 19/12/2017)	Mucopolissacaridose tipo II	83.442.677		
			5. alfagalsidase 1 mg/ml	Não incorporado (decisão de não incorporação Portaria nº 56, de 23/11/2020)	Doença de Fabry	80.920.978		
			6. atalureno 250 mg	Não incorporado (sem avaliação pela Conitec)	Distrofia Muscular de Duchenne	55.633.789		
			7. beta-agalsidase 35 mg	Não incorporado (decisão de não incorporação Portaria nº 56, de 23/11/2020)	Doença de Fabry	37.717.391		
			8. metreleptina 11,3 mg	Não incorporado e sem registro na Anvisa	Lipodistrofia generalizada Congênita ou adquirida	32.054.438		
			9. lomitapida 10 mg	Não incorporado (sem avaliação pela Conitec)	Hipercolesterolemia familiar	23.927.105		
			10. inibidor de esterase C1 humana 500 UI	Não incorporado (sem avaliação pela Conitec)	Angioedema hereditário tipo I e II	22.945.735		
2017	508	1.085.490.298	1. eculizumabe 10 mg/ml	2018 (Portaria nº 77, de 14/12/2018)	Hemoglobinúria paroxística noturna	296.462.726	981.720.251	90,4
			2. galsulfase 1 mg/ml	2018 (Portaria nº 83, de 19/12/2018)	Mucopolissacaridose tipo VI	176.252.001		
			3. elosulfase alfa 1 mg/ml	2018 (Portaria nº 82, de 19/12/2018)	Mucopolissacaridose tipo IVa (Síndrome de Morquio A)	101.093.948		
			4. alfagalsidase 1 mg/ml	Não incorporado (decisão de não incorporação Portaria nº 56, de 23/11/2020)	Doença de Fabry	100.592.156		
			5. atalureno 250 mg	Não incorporado (sem avaliação pela Conitec)	Distrofia Muscular de Duchenne	84.746.590		
			6. idursulfase 2 mg/ml	2017 (Portaria nº 62, de 19/12/2017)	Mucopolissacaridose tipo II	61.684.075		
			7. beta-agalsidase 35 mg	Não incorporado (decisão de não incorporação Portaria nº 56, de 23/11/2020)	Doença de Fabry	54.882.175		
			8. atalureno 1000 mg	Não incorporado (sem avaliação pela Conitec)	Distrofia Muscular de Duchenne	39.848.443		
			9. alfa-alglicosidase 50 mg	2019 (Portaria nº 48, 16/10/2019)	Doença de Pompe	33.258.882		
			10. metreleptina 11,3 mg	Não incorporado e sem registro na Anvisa	Lipodistrofia generalizada congênita ou adquirida	32.899.255		
2018	332	1.495.905.549	1. eculizumabe 10 mg/ml	2018 (Portaria nº 77, de 14/12/2018)	Hemoglobinúria paroxística noturna	478.746.855	1.364.024.905	91,2
			2. galsulfase 1 mg/ml	2018 (Portaria nº 83, de 19/12/2018)	Mucopolissacaridose tipo VI	164.038.893		
			3. idursulfase 2 mg/ml	2017 (Portaria nº 62, de 19/12/2017)	Mucopolissacaridose tipo II	124.079.026		
			4. nusinersena 2,4 mg/ml	2021 (Portaria nº 26, de 1/6/2021)	Atrofia muscular espinhal 5q tipo II, com diagnóstico até os 18 meses de idade	124.045.961		
			5. elosulfase alfa 1 mg/ml	2018 (Portaria nº 82, de 19/12/2018)	Mucopolissacaridose tipo IVa (Síndrome de Morquio A)	112.287.809		
			6. alfagalsidase 1 mg/ml	Não incorporado (decisão de não incorporação Portaria nº 56, de 23/11/2020)	Doença de Fabry	105.496.952		
			7. atalureno 250 mg	Não incorporado (sem avaliação pela Conitec)	Distrofia Muscular de Duchenne	100.947.361		
			8. atalureno 1000 mg	Não incorporado (sem avaliação pela Conitec)	Distrofia Muscular de Duchenne	55.002.927		
			9. beta-agalsidase 35 mg	Não incorporado (decisão de não incorporação Portaria nº 56, de 23/11/2020)	Doença de Fabry	53.825.639		
			10. alfa-alglicosidase 50 mg	2019 (Portaria nº 48, 16/10/2019)	Doença de Pompe	45.553.481		
2019	284	1.004.114.249	1. eculizumabe 10 mg/ml	2018 (Portaria nº 77, de 14/12/2018)	Hemoglobinúria paroxística noturna	467.571.795	950.325.051	94,6
			2. elosulfase alfa 1 mg/ml	2018 (Portaria nº 82, de 19/12/2018)	mucopolissacaridose tipo IVa (Síndrome de Morquio A)	190.076.099		
			3. alfagalsidase 1 mg/ml	Não incorporado (decisão de não incorporação Portaria nº 56, de 23/11/2020)	Doença de Fabry	105.003.061		
			4. nusinersena 2,4 mg/ml	2021 (Portaria nº 26, de 1/6/2021)	Atrofia muscular espinhal 5q tipo II	87.703.893		
			5. galsulfase 1 mg/ml	2018 (Portaria nº 83, de 19/12/2018)	Mucopolissacaridose tipo VI	54.288.525		
			6. metreleptina 11,3 mg	Não incorporado e sem registro na Anvisa	Lipodistrofia generalizada congênita ou adquirida	21.533.960		
			7. eteplirsena 50 mg/ml	Não incorporado e sem registro na Anvisa	Distrofia Muscular de Duchenne	8.324.480		
			8. sebelipase alfa 2 mg/ml	Não incorporado (sem avaliação pela Conitec)	Deficiência de lipase ácida lisossomal	6.308.632		
			9. brentuximabe vedotina 50 mg	2019 (Portaria nº 12, de 11/3/2019)	Linfoma de hodgkin cd30+	5.976.991		
			10. mercaptamina 75 mg	Não incorporado e sem registro na Anvisa	Cistinose nefropática	3.537.616		
2020	678	592.320.679	1. eculizumabe 10 mg/ml	2018 (Portaria nº 77, de 14/12/2018)	Hemoglobinúria paroxística noturna	220.640.260	484.460.069	81,8
			2. atalureno 250 mg	Não incorporado (sem avaliação pela Conitec)	Distrofia Muscular de Duchenne	107.559.900		
			3. clozapina 100 mg	2015 (Portaria nº 3, de 9/3/2015) e 2016 (Portaria nº 22, de 31/5/2016)	Transtorno afetivo bipolar e psicose associada à doença de Parkinson	42.825.048		
			4. metreleptina 11,3 mg	Não incorporado e sem registro na Anvisa	Lipodistrofia generalizada congênita ou adquirida	36.465.099		
			5. alfa-alglicosidase 50 mg	2019 (Portaria nº 48, 16/10/2019)	Doença de Pompe	19.945.651		
			6. atalureno 1000 mg	Não incorporado (sem avaliação pela Conitec)	Distrofia Muscular de Duchenne	19.580.225		
			7. atalureno 125 mg	Não incorporado (sem avaliação pela Conitec)	Distrofia Muscular de Duchenne	15.212.043		
			8. mercaptamina 75 mg	Não incorporado e sem registro na Anvisa	Cistinose nefropática	10.014.300		
			9. sebelipase alfa 2 mg/ml	Não incorporado (sem avaliação pela Conitec)	Deficiência de lipase ácida lisossomal	8.655.498		
			10. cerliponase alfa 30 mg/ml	Em análise pela Conitec	Lipofuscinose ceroide neuronal tipo 2	3.562.045		

Conitec: Comissão Nacional de Incorporação de Tecnologias no Sistema Único de Saúde.

Fontes: i) Sistema Integrado de Administração de Serviços Gerais (SIASG). Relatório disponibilizado por técnico do Ministério da Saúde; ii) Conitec. Funcionalidades: Tecnologias Demandadas e Recomendações da Conitec. Disponível em: <http://conitec.gov.br/>. Acesso em: 25 jan 2022.

Nota: o mesmo fármaco, mas em concentrações diferentes, foi contabilizado como dois ou mais medicamentos. Exemplo: atalureno.

O gasto do Ministério da Saúde em ações judiciais de medicamentos cresceu significativamente entre 2012 e 2016 (221%), chegando a R$ 1,5 bilhão no último ano. Decresceu 26% entre 2016 e 2017, e manteve-se no patamar de R$ 1,1 bilhão de 2017 a 2019 (Figura 2).

**Figura 2:** Gasto do Ministério da Saúde em ações judiciais de medicamentos e com o Componente Especializado da Assistência Farmacêutica - CEAF (2012–2020).

Fonte: Siga Brasil. Disponível em: <https://bit.ly/3g50ClC>. Acesso em: 25 jan 2022.

Nota: Gasto em ações judiciais se refere à despesa liquidada, registrada na Unidade Gestora Responsável 250925-SJ-MED/MS. Gasto com o CEAF diz respeito à despesa liquidada, registrada na ação orçamentária 4705–Apoio Financeiro para Aquisição e Distribuição de Medicamentos do Componente Especializado da Assistência Farmacêutica.

Estudos realizados nos anos 2000 já evidenciavam preocupação com as consequências das demandas judiciais. Questões como a desconsideração das responsabilidades dos entes da federação na organização do SUS, a compra de medicamentos não incorporados e sem registro na Anvisa, além de prejuízos à equidade, foram explicitadas^12^^,^^13^.

Na última década, autores vinculados ao sistema de justiça entraram no debate, destacando aspectos positivos e negativos da judicialização da saúde, o que representa um avanço por favorecer a discussão sobre o tema entre seus pares.

Quanto aos aspectos positivos, destacam-se o fomento à formulação e à revisão de políticas públicas, a inclusão da saúde na agenda política, o desenvolvimento da avaliação de tecnologias em saúde e a ampliação do diálogo entre os poderes^14^^,^^15^.

Com relação aos aspectos negativos, ressaltam-se a desorganização do SUS e das finanças públicas, as escolhas judiciais indevidas de políticas públicas, a fragilização da isonomia, a desconsideração dos critérios de priorização das tecnologias disponibilizadas e a ampliação das desigualdades em saúde^3^^,^^4^^,^^15^.

Além disso, permanece o entendimento de que o Judiciário pode considerar evidências científicas e determinar o fornecimento de tecnologias não incorporadas ao SUS, em um processo paralelo e capilarizado em todo o país, que concorre com a avaliação de tecnologias realizada pelo sistema de saúde^1^.

Esse entendimento tem várias implicações. Salientam-se duas delas: i) a fragilização das políticas como meio para a garantia do direito à saúde, pois, com tantas exceções à Relação Nacional de Medicamentos Essenciais (Rename), desvirtua-se a regra, que deveria valer para todos; e ii) a redução dos recursos previstos no orçamento do ano para garantir o acesso da população aos medicamentos da Rename. Esta segunda implicação é discutida a seguir.

### Reserva do Possível, Judicialização e Orçamento Público

Sobre a relação entre o orçamento público, a judicialização da saúde e a teoria da reserva do possível, as manifestações de parte dos gestores públicos e dos magistrados se contrapõem. De um lado, gestores se utilizam dessa teoria para justificar a impossibilidade de atendimento da demanda judicial, alegando a indisponibilidade de recursos para atendê-la. De outro lado, juízes contra-argumetam que não se pode sobrepor um interesse secundário do Estado ao direito à saúde sob o argumento da reserva do possível. O fato é que ambas as posições precisam ser repensadas.

O conceito de reserva do possível foi importado da Alemanha, onde houve reconhecimento de que questões envolvendo direitos sociais sofrem limitação em três dimensões: i) reserva do faticamente possível: a satisfação da demanda precisa ser viável; ii) reserva do juridicamente possível: a demanda precisa ser juridicamente possível; e iii) reserva do financeiramente possível: o atendimento à demanda fica limitado à capacidade financeira do Estado^16^.

Em relação ao uso da teoria da reserva do possível pelos gestores, ressalta-se que não se pode confundir capacidade financeira do Estado com o orçamento anual estabelecido para a saúde. A capacidade financeira do Estado é medida considerando todos os recursos que são arrecadados da sociedade. É claro que a capacidade de gasto com políticas que concretizam direitos sociais é limitada pelas receitas, sendo fundamental a participação da sociedade na discussão conduzida por seus representantes sobre a destinação dos recursos^17^. Mas não se pode alegar escassez de recursos considerando apenas o orçamento anual da saúde.

Quanto ao contra-argumento dos magistrados, tampouco se pode considerar a capacidade financeira um interesse secundário do Estado. Os direitos têm custos, sendo preciso mensurar qual é o seu custo e definir como serão financiados, assim como controlar quem decide sobre os recursos que serão alocados para concretizá-los^18^.

Quando o Judiciário ignora as macroquestões relacionadas ao tema e determina o fornecimento, para um indivíduo, de medicamentos não previstos nas políticas públicas, ele impacta o acesso dos demais aos medicamentos que constam nas políticas^19^. Isso ocorre porque o orçamento tem natureza de planejamento e é definido no ano anterior ao de sua vigência. A realocação de recursos entre diferentes áreas requer prévia autorização legislativa, logo, não se trata da simples vontade do gestor da saúde^20^ e, a depender das condições econômicas do país, a restrição orçamentária pode implicar a realização de escolhas trágicas na oferta de bens e serviços.

As Figuras 2 e 3 ilustram bem esse tipo de problema. No Ministério da Saúde, as despesas com ações judiciais de medicamentos são financiadas, desde 2014, exclusivamente por recursos alocados na ação orçamentária que financia o Componente Especializado da Assistência Farmacêutica (CEAF). Considerando apenas o gasto registrado nesta ação, observa-se que a participação das despesas por demandas judiciais cresceu desde 2012, chegando a 25,2% em 2019. Com isso, restam menos recursos para financiar o elenco de medicamentos desse componente para toda a população.

**Figura 3:** Participação do gasto do Ministério da Saúde em ações judiciais de medicamentos no gasto total com o Componente Especializado da Assistência Farmacêutica (2012–2020).

Fonte: Siga Brasil. Disponível em: <https://bit.ly/3g50ClC>. Acesso em: 25 jan 2022.

Nota: despesa liquidada registrada na Unidade Gestora Responsável 250925-SJ-MED/MS.

Analisando-se a Tabela e a Figura 2, observa-se que apenas 10 medicamentos foram responsáveis por 94,6% do gasto em ações judiciais do Ministério da Saúde em 2019 (R$ 950,33 milhões) e consumiram 21% dos recursos destinados ao CEAF. Com essa situação, não surpreende o relato de falta de medicamentos que estão sob a responsabilidade de aquisição do Ministério da Saúde^21^^,^^22^, que deve ter diversos determinantes, mas certamente um deles é a judicialização.

Esse exemplo explicita de que forma a judicialização pode causar mais danos do que promover o direito à saúde no Brasil. Decisões sobre alocação de recursos são complexas e demandam legitimidade democrática. Ademais, requerem sólido conhecimento técnico em saúde. Não podem ter por único fundamento critérios que denotam sentimentos humanitários de solidariedade, justiça e empatia para com um único indivíduo^23^. Isso acaba levando a decisões que desconsideram a legislação sanitária do país e que colocam em risco a saúde das pessoas, como no caso da fosfoetanolamina^24^. Mas, enfim, de que forma o Judiciário poderia melhor proteger o direito à saúde?

### Necessidade de Novos Rumos

Não há dúvidas de que o Judiciário exerce importante papel quando determina que o Estado cumpra o seu dever de garantir o fornecimento de medicamentos incorporados ao SUS, em observância às diretrizes e normativos das políticas públicas. Diretrizes e normativos estes que 46% dos magistrados, em pesquisa recente, disseram não observar^6^.

Enquanto isso, decisões de política fiscal são tomadas no âmbito da União com grande impacto negativo nos direitos sociais, como a implantação do teto de gastos para as despesas primárias, o congelamento das aplicações mínimas em saúde e educação, a ausência de limitação das despesas financeiras e a ampliação do gasto tributário^25^.

Dada a importância da política fiscal para a garantia de direitos, discutem-se hoje princípios de direitos humanos para a política fiscal, em uma iniciativa que busca aproximar os campos da economia e do direito^26^. O Judiciário precisa exercitar a macrojustiça, o que demanda controle sobre processos envolvendo as políticas macroeconômicas que afetam o custeio do SUS^1^^,^^27^. Caso não a exerça, continuará fomentando iniquidades em saúde.

Dessa forma, é necessário que se reflita sobre o papel do Judiciário na proteção dos direitos sociais. Segundo Ferraz^28^ (2011), eles seriam adequadamente protegidos se o Judiciário parasse de interferir no conteúdo das políticas e passasse a atuar no controle da sua formulação para garantir o respeito às normas constitucionais e legais. Essa é a mesma posição de Gebran Neto^15^ (2019).

Assim, não se questiona a atuação do Judiciário na garantia do acesso aos medicamentos incorporados ao SUS, respeitadas as normas estabelecidas no âmbito do sistema de saúde. Se as normas e os procedimentos são questionáveis, considerando-se as balizas da Constituição Federal de 1988 e das leis, discutem-se as normas e os procedimentos. Mas não são razoáveis as alocações de recursos por ações judiciais para a compra de medicamentos não incorporados. Essas destinações descaracterizam a regra geral que deveria ser aplicada para todos e drenam recursos escassos, enquanto o SUS é submetido a um processo de subfinanciamento crônico^29^^,^^30^. Dessa forma, a universalidade fica prejudicada e o direito à saúde permanece sendo negado aos mais desfavorecidos socioeconomicamente.
